# Supplementary material for: Evolutionary and structural analyses of SARS-CoV-2 D614G spike protein mutation now documented worldwide
Source: Sci Rep. 2020 Aug 20;10:14031. doi: 10.1038/s41598-020-70827-z (PMC7441380; doi:10.1038/s41598-020-70827-z)
Supplement: Supplementary file 1 [file 41598_2020_70827_MOESM1_ESM.docx]

| **Country** | **n = D** | **n = G** | **Total n** | **% G** |
| --- | --- | --- | --- | --- |
| **Algeria** | 0 | 2 | 2 | 100% |
| **Australia** | 56 | 8 | 64 | 13% |
| **Belgium** | 9 | 81 | 90 | 90% |
| **Brazil** | 6 | 29 | 35 | 83% |
| **Cambodia** | 1 | 0 | 1 | 0% |
| **Canada** | 46 | 34 | 80 | 43% |
| **Chile** | 6 | 1 | 7 | 14% |
| **China** | 310 | 4 | 314 | 1% |
| **Colombia** | 1 | 1 | 2 | 50% |
| **Congo (Kinshasa)** | 2 | 17 | 19 | 89% |
| **Czechia** | 0 | 3 | 3 | 100% |
| **Denmark** | 0 | 9 | 9 | 100% |
| **Ecuador** | 1 | 0 | 1 | 0% |
| **Finland** | 8 | 32 | 40 | 80% |
| **France** | 16 | 103 | 119 | 87% |
| **Georgia** | 5 | 5 | 10 | 50% |
| **Germany** | 15 | 11 | 26 | 42% |
| **Greece** | 1 | 2 | 3 | 67% |
| **Hungary** | 0 | 3 | 3 | 100% |
| **Iceland** | 71 | 271 | 342 | 79% |
| **India** | 2 | 0 | 2 | 0% |
| **Ireland** | 4 | 9 | 13 | 69% |
| **Italy** | 3 | 20 | 23 | 87% |
| **Japan** | 82 | 2 | 84 | 2% |
| **Korea, South** | 13 | 0 | 13 | 0% |
| **Kuwait** | 4 | 0 | 4 | 0% |
| **Lithuania** | 0 | 1 | 1 | 100% |
| **Luxembourg** | 1 | 9 | 10 | 90% |
| **Malaysia** | 10 | 0 | 10 | 0% |
| **Mexico** | 0 | 1 | 1 | 100% |
| **Nepal** | 1 | 0 | 1 | 0% |
| **Netherlands** | 84 | 106 | 190 | 56% |
| **New Zealand** | 6 | 2 | 8 | 25% |
| **Nigeria** | 0 | 1 | 1 | 100% |
| **Norway** | 7 | 1 | 8 | 13% |
| **Pakistan** | 1 | 0 | 1 | 0% |
| **Panama** | 0 | 1 | 1 | 100% |
| **Peru** | 0 | 1 | 1 | 100% |
| **Poland** | 1 | 0 | 1 | 0% |
| **Portugal** | 6 | 38 | 44 | 86% |
| **Russia** | 0 | 1 | 1 | 100% |
| **Saudi Arabia** | 1 | 2 | 3 | 67% |
| **Senegal** | 1 | 11 | 12 | 92% |
| **Singapore** | 14 | 0 | 14 | 0% |
| **Slovakia** | 1 | 3 | 4 | 75% |
| **South Africa** | 0 | 1 | 1 | 100% |
| **Spain** | 22 | 18 | 40 | 45% |
| **Sweden** | 1 | 0 | 1 | 0% |
| **Switzerland** | 2 | 50 | 52 | 96% |
| **Taiwan*** | 16 | 2 | 18 | 11% |
| **Thailand** | 2 | 0 | 2 | 0% |
| **Turkey** | 1 | 0 | 1 | 0% |
| **United Kingdom** | 242 | 183 | 425 | 43% |
| **US** | 471 | 155 | 626 | 25% |
| **Vietnam** | 5 | 3 | 8 | 38% |
| **Total** | **1558** | **1237** | **2795** | **44%** |

**Supplementary data Table 1. Country Distribution of SARS-CoV-2 Genome Sequences Possessing the Spike Protein D614G Mutation.** The number of sequences with either D (n = D) or G (n = G) amino acid at position 614 of the spike protein and total (n) per country, and the percent (%) of G from each country are presented.
