# Supplementary material for: Evolutionary and structural analyses of SARS-CoV-2 D614G spike protein mutation now documented worldwide
Source: Sci Rep. 2020 Aug 20;10:14031. doi: 10.1038/s41598-020-70827-z (PMC7441380; doi:10.1038/s41598-020-70827-z)
Supplement: Supplementary file 2 [file 41598_2020_70827_MOESM2_ESM.docx]

| **Age group (yrs)** | **Case Fatality Rate by country (%)** | | | | | **Spearman’s rank correlation r** | **P-value** |
| --- | --- | --- | --- | --- | --- | --- | --- |
|  | **China as of 11 Feb 2020** | **Italy as of 17 March 2020** | **South Korea as of 12 March 2020** | **Spain as 10 April 2020** | **Canada as of 9 April 2020*** |  |  |
| ≥ 80 | 14.8 | 20.2 | 8.5 | 21.5 | 12.3 | 0.80 | 0.33 |
| 70-79 | 8 | 12.8 | 5.0 | 10.7 | 2.8 | 1.00 | 0.08 |
| 60-69 | 3.6 | 3.5 | 1.5 | 3.4 |  | 0.40 | 0.75 |
| 50-59 | 1.3 | 1 | 0.4 | 1.0 | 0.3 | 0.32 | >0.99 |
| 40-49 | 0.4 | 0.4 | 0.1 | 0.4 |  | 0.77 | 0.50 |
| 30-39 | 0.2 | 0.3 | 0.1 | 0.2 | 0.1 | 0.95 | 0.17 |
| 20-29 | 0.2 | 0 | 0 | 0.2 |  | 0 | >0.99 |
| 10 to 19 | 0.2 | 0 | 0 | 0.2 | 0 | 0 | >0.99 |
| 0-9 | 0 | 0 | 0 | 0.3 |  | 0.26 | >0.99 |
| All ages | 2.3 | 7.2 | 0.9 | 6.5 | 2.3 | 1.00 | 0.08 |
|  | **Percent of G (%)** | | | | |  |  |
| All ages | 1.2 | 86.9 | 0 | 45.0 | 42.5 | - | - |

**Supplementary data Table 2. Crude Fatality Rate by age and percent of D614G mutation for China, Italy, South Korea, Spain and Canada.** Correlation between CFR and the percent of G at position 614 of the spike protein was calculated using Spearman’s rank correlation. *Canadian data presented in 20 years ranges were excluded from Pearson correlation analysis.
